# Study on stripe rust (*Puccinia striiformis*) effect on grain filling and seed morphology building of special winter wheat germplasm Huixianhong

**DOI:** 10.1371/journal.pone.0215066

**Published:** 2019-05-21

**Authors:** Chunyu He, Yanhong Zhang, Wei Zhou, Qingyi Guo, Bin Bai, Sanbao Shen, Gaobao Huang

**Affiliations:** 1 Pharmacy Department of Gansu University of Chinese Medicine, Lanzhou, Gansu, China; 2 Agronomy Department of Gansu Agricultural University, Gansu Provincial Key Laboratory of Arid Land Crop Science, Lanzhou, Gansu, China; 3 Division of Agriculture, Dept. of Horticulture 316 PTSC, University of Arkansas, Fayetteville, Arkansas, United States of America; 4 Wheat Institute of Gansu Agricultural Science Academy, Lanzhou, Gansu, China; 5 Qinshui Station for Popularizing Agricultural Technique, Tianshui, Gansu, China; Murdoch University, AUSTRALIA

## Abstract

Stripe rust, caused by *Puccinia striiformis f*. *sp*. *tritici* (Pst), is an airborne fungal disease which always destructs leaf and leads to stagnation of grain filling, decreasing of kernel weight, thin seed and yield loss. Winter wheat Huixianhong is a special germplasm with special characteristics of tolerance or resistance against stripe rust. In order to understand the effect on Huixianhong from stripe rust, we designed two kinds of treatment, inoculation of stripe rust fungi (IH) and artificial immune by bactericide (CK) to study the dynamic of disease process, the grain filling and the thousand-kernel weight (TKW). Our results showed that the incubation period of Hongxinahui was 13.5 days. The prevalence increased from 32.9% at 15 days after jointing to 80.0% at 9 days after booting, and reached to 97.0% at the heading stage. The infection type (IT) was 7 to 9 at the beginning of anthesis. The severity, leaf withered area ratio and disease index at the 15^th^ day after anthesis were 67.17%, 98.17% and 0.6717, respectively. The IH maximum increasing rate of leaf necrosis and chlorosis area was from heading beginning stage to anthesis beginning stage, which increased from 18.66 mm^2^·d^-1^ to 21.04 mm^2^·d^-1^. The maximum rate of grain filling was 1.25 mg·d^-1^ at the 18^th^ day after anthesis, which was earlier than that of CK by 3.3 days. The IH thousand-kernel weight Loss (TKWL) was more than that of CK by 6.19%, the stage of heading and amature were 3.0 days and 4.5 days earlier than CK, respectively. The stripe rust infection seriously destructed the photosynthetic function of leaf at the earlier stage of grain filling, i.e. at the beginning of anthesis, which led to the most important biomass loss and the grain filling rate decrease. As far as stripe rust is concerned, Huixianhong is a high susceptible, easily been infected, seriously showing symptoms and most quickly epidemic type but can successfully complete grain filling in high quality. It is a very useful germplasm for creating and selecting special breeding materials against stripe rust.

## Introduction

Epidemics of wheat stripe rust, caused by *Puccinia striiformis* f. sp. *tritici*, are more frequent around the world and often led to serious yield loss while applying for the resistance wheat cultivar or materials can significantly decrease leaf damage and yield loss. So how to find and breed excellent wheat materials and cultivar is very important for wheat breeding and production. The winter wheat cultivar Huixianhong is the typical one with more excellent characteristics and been found by Dr. Chunyu He.

The stripe rust (*Puccinia striiformis*) (Pst) always seriously destructs photosynthetic function of leaf and affects supply and movement of assimilation products, which leads to thousand-kernel weight (TKW) decrease and yield loss[1,2]. Huixianhong is a winter wheat cultivar that is high susceptible to stripe rust and epidemic as quickly as showing symptoms. The infection type (IT) can reach to 8–9 and the prevalence ratio and severity can arrive at 100% in a short time after symptoms. After infection, wheat leaves turn into chlorosis and lose photosynthetic function at the earlier stage of grain filling[2].

However, Huixianhong records from 1996 to 2010 year showed the thousand-kernel weight loss rate (TKWLR) usually were between 4.00% and 13.07% except for the two factors of severe drought and craze epidemic worked together could arrive at 24.04%-30.02%. The characteristics of keeping normal grain filling and decreasing TKWLR hardly has been reported by others[2–4].

The reported results about the grain filling and TKW most focused on the yield factors, compensation effect, pathology et al, while there was no report about keeping normal ability of graining filling and TKW when all leaves lost photosynthetic function [5,6]. In order to study on the relationship between the lower TKWLR and grain filling under the condition of lost leaf photosynthetic function, establish equation about the grain filling rate and seed morphology formation, the characteristic about the attack, developing, epidemic, damage of Pst and the grain filling characters were measured. So we hope through this study, we could provide theory and practice to keep normal yield when Pst was crazy epidemic.

## Materials and methods

### Cultivar and fungus

The original seed of Huixianhong has been provided by Germplasm Resource Bank of Gansu Agricultural Science Academy (GASA) and identified by professor Jiuyuan Du and Dr. Chunyu He. The cultivar has been purified and derived from one seed which has been identified as the pure line of Huixianhong by author, and has been propagated since 2005 year under control condition. The stripe rust (*Puccinia striiformis*) fungus has been supplied by Dr. Shiqin Cao from the Plant Protection Institute of GASA, which included the 12 main epidemic races at that time. The author can provide the seeds and methods if whoever want. The experiment field belongs to GASA and is opened for whoever wanted to carry out field trials or co-operate with them.

The immunization Triazolone wettable powder has been produced by Jiangsu Green Shield Plant protection Co., Ltd. The experimentation design was in random block design with three times repetition and the LSD and SSR of analysis of variance was used to put up multiple comparisons. The treatments have been designed in two types. One was artificial inoculation on Huixianhong (IH) which could assure completely incidence, another was applied for the triazolone wettable powder (TWP) to protect Huixianhong from being infected by the fungus (CK). The plot area was 81m^2^ (9m×9m) with 0.20 m spacing between rows, 0.03 m between plants and 0.40m between plots under manual spot seeding.

The inoculation on IH treatments has been done during the seedling stage on 2011, 2012 and 2013 year[7]. At the same time, CK treatments have been sprayed by TWP the first time and would be sprayed the other two times after 5 days and 10 days in order to assure CK from being infected by the fungus [2]. The susceptible Huixianhong was planted around the experiment plots to ensure sufficient inoculum for uniform disease infection.

The field trials performed at the Qinshui Experiment Station of GASA from 2011 to 2013 year, locating in Longnan Area of stripe rust serious epidemic area at latitude 34°45′, longitude 106°09′ and altitude 1430 m, which annual precipitation was 460–560 mm and growth stage precipitation was 231.2 mm during experiment, and annual frost-free period was 170 days.

The soil samples were tested based on the Standards of Determination of soil available Nitrogen (DB13/T 843–2007), soil total Nitrogen (NY/T 1121.24–2012), available Potassium in soil (DB13/T 844–2007) and total Phosphorus in soil (GB/T 9837–1988). The soil texture was sandy loam with organic matter 14.7mg·g^-1^, total nitrogen 0.9 mg·g^-1^, hydrolyzable nitrogen 67 mg·g^-1^, rapidly available phosphorus 12.3 mg·kg^-1^, rapidly available potassium 128.0 mg·kg^-1^, slowly available potassium 1.7 g·g^-1^ and the previous crop was winter wheat.

### Methods and Items

The measurements and recordings were the infected type (IT), disease severity, disease incidence, thousand-kernel weight (TKW), leaf necrosis and chlorosis area (IRLNC), leaf area (LA), weight of dry and fresh kernel, the increased area of necrosis and chlorosis under the fixed time and plant. The data based on the special formulas and mathematical models were the disease index (DI), grain filling rate (GFR), leaf withered area ratio (LWAR) and the increasing rate of leaf necrosis and chlorosis area (IRLNC) [2,7].

Infection type (IT) was based on a 0–9 scale, with 0 refers to no visible sporulation; 2 refers to necrotic and/or chlorotic stripes with no sporulation; 3 refers to necrotic and/or chlorotic stripes with only a trace of sporulation; 4, 5, and 6 refers to necrotic and/or chlorotic stripes with light, intermediate, and moderate sporulation, respectively; 7, 8, and 9 refers to abundant sporulation with necrotic and/or chlorotic stripes, chlorosis behind the sporulation area, and no chlorosis or necrosis, respectively [8]. On the basis of the 0–9 IT scale, accessions were grouped as highly resistant to moderately resistant (IT 0–3), intermediate (IT 4–6), and moderately susceptible to highly susceptible (IT 7–9). Disease severity was estimated visually as percentage of infected leaf area when rust severities on flag leaves of the susceptible check reached 80–100%[8].

The dynamic changes of stripe rust has been recorded since the 3^rd^ day after Jointing stage and kept recording each 3 days until fungus stop producing fresh spores and leaves turned into dried up.

### Samples of grain filling and mark Standards

The samples were single whole plant and single head which have been marked at the same anthesis and kept marking each 6 days after anthesis (Table 1). It was planned to take 25 marked heads each 6 days and chose 20 kernels from each head to study dried weight[2, 9,10].

**Table 1:** The abbreviation of growth stage and organs.

**Ab**.	Meaning	**Ab**.	Meaning
**IH**	inoculated Huixianhong	**Sh**_**1**_	the flag Sheath
**CK**	immunized Huixianhong	**Sh**_**2**_	the second-leaf sheath from top
**Sd**	seeds from single whole plant	**Sh**_**3**_	the third-leaf sheath from top
**L**_**1**_	flag leaf	**Sh**_**4**_	the other leaves’ sheaths except the upper three
**L**_**2**_	the second leaf from top	**Pl**	the time of planting
**L**_**3**_	the third leaf from top	**Sl**	the stage of seedling
**L**_**4**_	the other leaves except the upper three leaves	**Jo**	the stage of jointing
**S**_**1**_	the flag stem	**Bo**	the stage of booting
**S**_**2**_	the second-leaf stem from top	**He**	the stage of heading
**S**_**3**_	the third-leaf stem from top	**An**	the stage of anthesis
**S**_**4**_	the other leaves’ stems except the upper three	**GF**	the stage of grain filling
**0d**	the beginning of some stage	**An0d**	the beginning of anthesis
**3d**	the 3 days after some stage	**An3d**	the 3 days after anthesis

Note: The table detailed and introduced the abbreviation of different growth stages and different organs.

### Data analysis

The data has been analyzed and the models have been built by the software of SPSS 19.0 and EXCELL 2007 [11].

## Results

### 1 The characteristics of leaf stress reaction under Pst infection

#### 1.1 The characteristics of incubation period

From the first time of inoculation to the symptom stage, the incubation periods were 15.5 days、13.0 days and12.0 days in 2011 year, 2012year and 2013year, respectively, and the three-year average was 13.5 days. At 9 days after jointing stage (Jo9d), there were necrotic and/or chlorotic stripes with light sporulation on L_1_, L_2_ and L_3_ and infection type (IT) was 2–4. At booting stage (Bo), uredinium on the above three leaves became larger and IT was 4–7. And there was light uredinium also appeared on L_4_. There was zero on CK leaves, which showed immunization was perfect.

#### 1.2 The three-year average dynamic changes of prevalence (DCP) from 2011 to 2013 years

**Fig 1:**
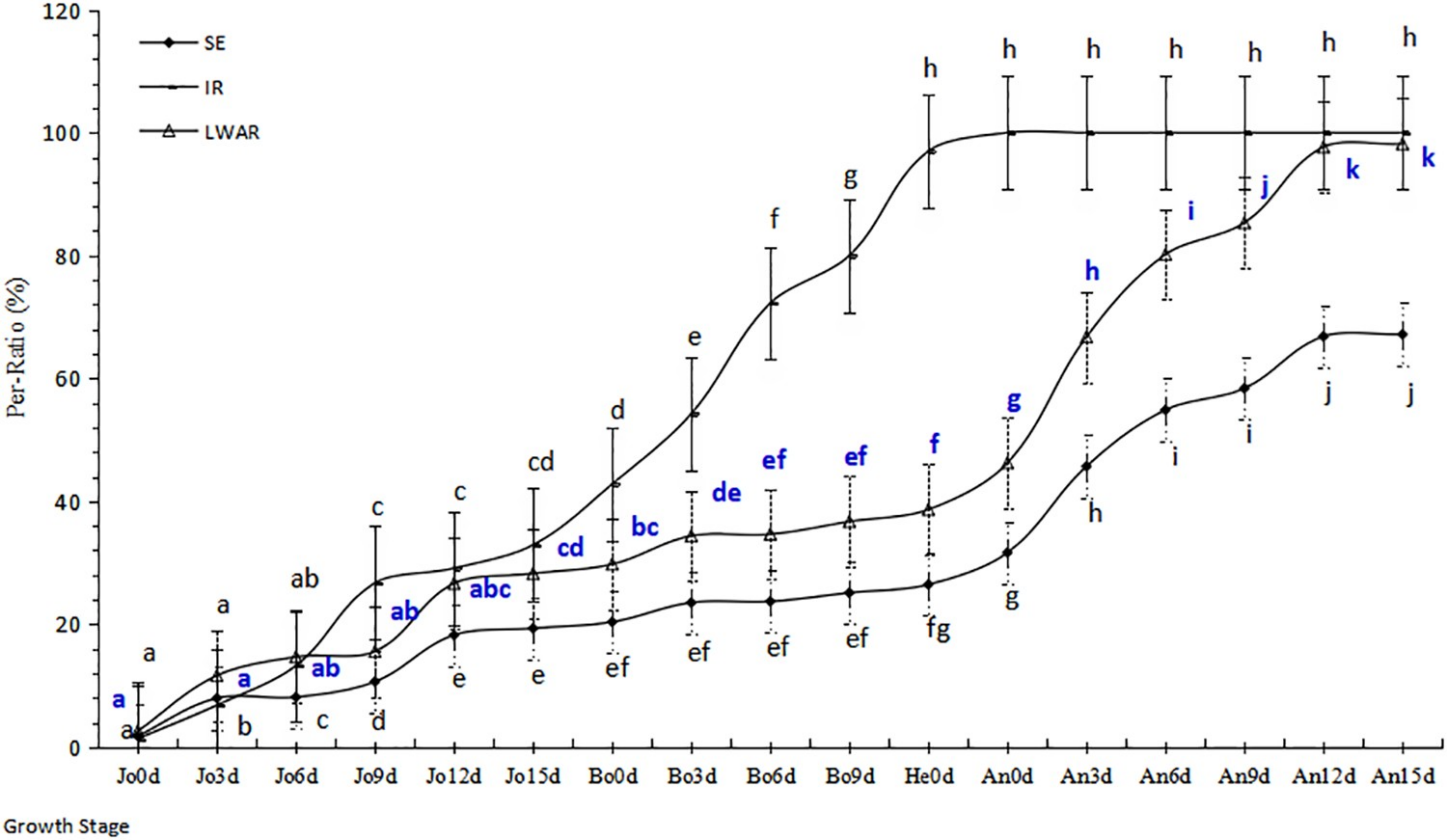
Dynamic of developing and damage. Note: 1) The letters of the dynamic line mean there were significant differences at the level of 0.05, while the same letters mean no difference. 2) GS means growth stages. As is the follows.

The three-year average dynamic changes of prevalence from 2011 to 2013 years behaved in S-shaped characters (Fig 1, S1 Table). The prevalence rate increased slowly at the beginning after jointing, extended quickly at mid-stage of anthesis, and decreased at the end of anthesis. The prevalence was 6.80% and characterized by L_1_ of IT 1–3 from symptom appearance at Jo3d.

The number of infected plants increased significantly from 13.25% Jo6d to 32.90% Jo15d (P<0.01). L_1_, L_2_ and L_3_ were all infected, necrotic and/or chlorotic stripes increased significantly, too. And L_4_ also appeared light symptom.

At the stage from Bo0d to Bo9d, the rate of infected plants increased most quickly, the prevalence quickly extended significantly from 42.80% to 80.00% (P<0.01). The necrotic and/or chlorotic stripes of L_1_, L_2_, L_3_ and L_4_ were characterized by large connected sheets and stripes, so did the abundant fresh uredinium.

At the heading stage, 97.00% plants had been infected by Pst and chlorotic stripes increased quickly and turned necrotic sheets during short time.

At An15d, the senescence rate of L_1_, L_2_ and L_3_ was 85.00% to 95.00% and L_4_ was 100.00%. But at the same time, the green area of the stems and sheaths kept 100.00% and heads were 70.10%. At the prior to middle stage of grain filling, the prevalence arrived at 100.00%.

#### 1.3 The three-year average dynamic changes of severity (DCS) from 2011 to 2013 years

The severity increased slowly from the beginning of jointing (Jo0d) to booting (Bo0d) while from An0d to An15d it extraordinarily significantly increased by112.3% (P<0.01) from 31.64% to 67.17%. After An15d, the infection rate of urediospore became slowly and the number of fresh uredium also decreased significantly, so did the increasing rate of Severity.

#### 1.4 The three-year average dynamic changes of disease index (DI) from 2011 to 2013 years

**Fig 2:**
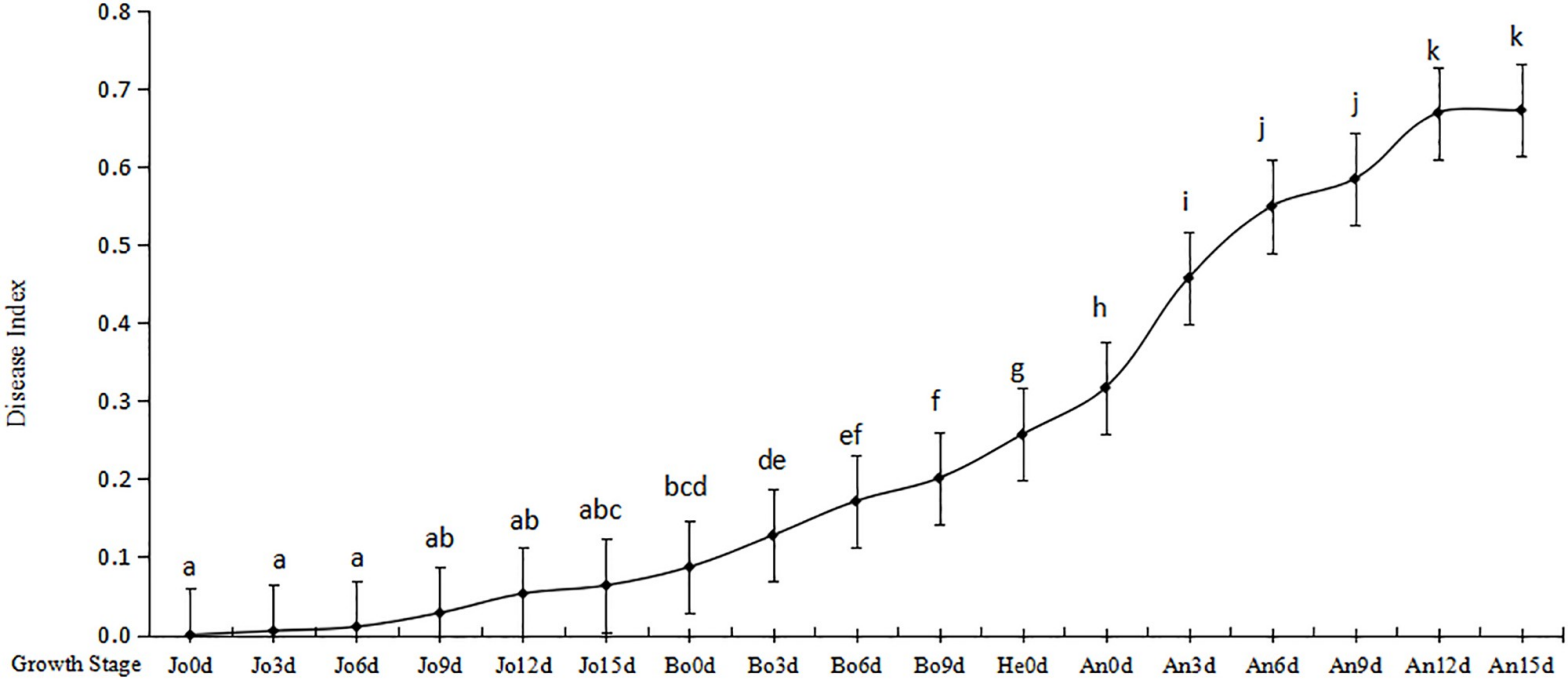
Dynamic of disease index. Note: The letters of the dynamic line means there were significant differences at the level of 0.05.

The results showed the disease index (DI) was less than 0.009 before Bo0d (Fig 2, S1 Table), which hardly could infect normal photosynthesis. From Bo3d to He, DI significantly increased from 0.1275 to 0.2566(P<0.05) while at An0d significantly arrived at 0.3164(P<0.01). From An0d to An15d, DI increased extraordinarily significantly from 0.3164 to 0.6717 (P<0.01) and kept increasing characteristics at the grain filling until lost photosynthetic function.

#### 1.5 The three-year average dynamic changes of leaf withered area ratio (LWAR) from 2011 to 2013 years

From Bo0d to Bo9d (Fig 1, S1 Table), LWAR of L_1_ significantly increased from 2.60% to 15.56% (P<0.05), necrosis and chlorosis areas appeared together along the leaf veins, whose IT was 1–3 and uredinium was small. At the stage, the disease on L_1_ was more serious than that of L_2_ and L_3_.

From Jo12d to He (Fig 1, S1 Table), LWAR increased significantly from 26.63% to 38.65% (P<0.05) and IT was 4–6. The uredinium characterized by dotted distribution was a little large and could be distinguished by eyes. The necrosis and chlorosis around the uredinium characterized by short narrow stripe and dotted shape had been enlarging along the leaf mains and appeared together on leaf. The L_1_ and L_2_ were infected more serious than that of L_3_ while L_4_ hardly could see the necrosis and chlorosis made by the uredinium.

As stepping into the blooming stage (Fig 1, S1 Table), the temperature, humidity and precipitation were beneficial to stripe rust epidemic meanwhile the number of uredinium accumulation also was large enough to produce more harmful to leaf and destruct photosynthesis function. The LWAR increased extraordinarily significantly from 46.23% at An0d to 98.17% at An15d (P<0.01). The IT was 7–9 and uredinium on L_1_, L_2_ and L_3_ characterized by stripe and innumerable piled spores were most seriously, which blocked photosynthesis and led to large withered areas, then leaves were dead.

After An15d (Fig 1, S1 Table), most golden uredinum turned into brown teleutodonidium and became into the oversummer stage when the temperature was higher than 26°C. At the same stage, leaf photosynthetic function had been destroyed absolutely while the stems, sheaths and glum were still in visible green. Although the leaf photosynthetic function had been lost for the stripe rust at the most important grain filling stage, the cultivar function of grain filling still kept normally until the kernel plumpness at harvest stage.

The LWAR was significantly difference between the stages of Jo0d~Jo9d, Jo12d~He and An0d~An15d (P<0.05) (Fig 1, S1 Table). Huixianhong is a special winter wheat cultivar characterized by high susceptible, rapid epidemic and most serious infected type but the function of grain filling keeping normal.

#### 1.6 The three-year average dynamic changes of the increasing rate of leaf necrosis and chlorosis area (IRLNC) from 2011 to 2013 years

**Fig 3:**
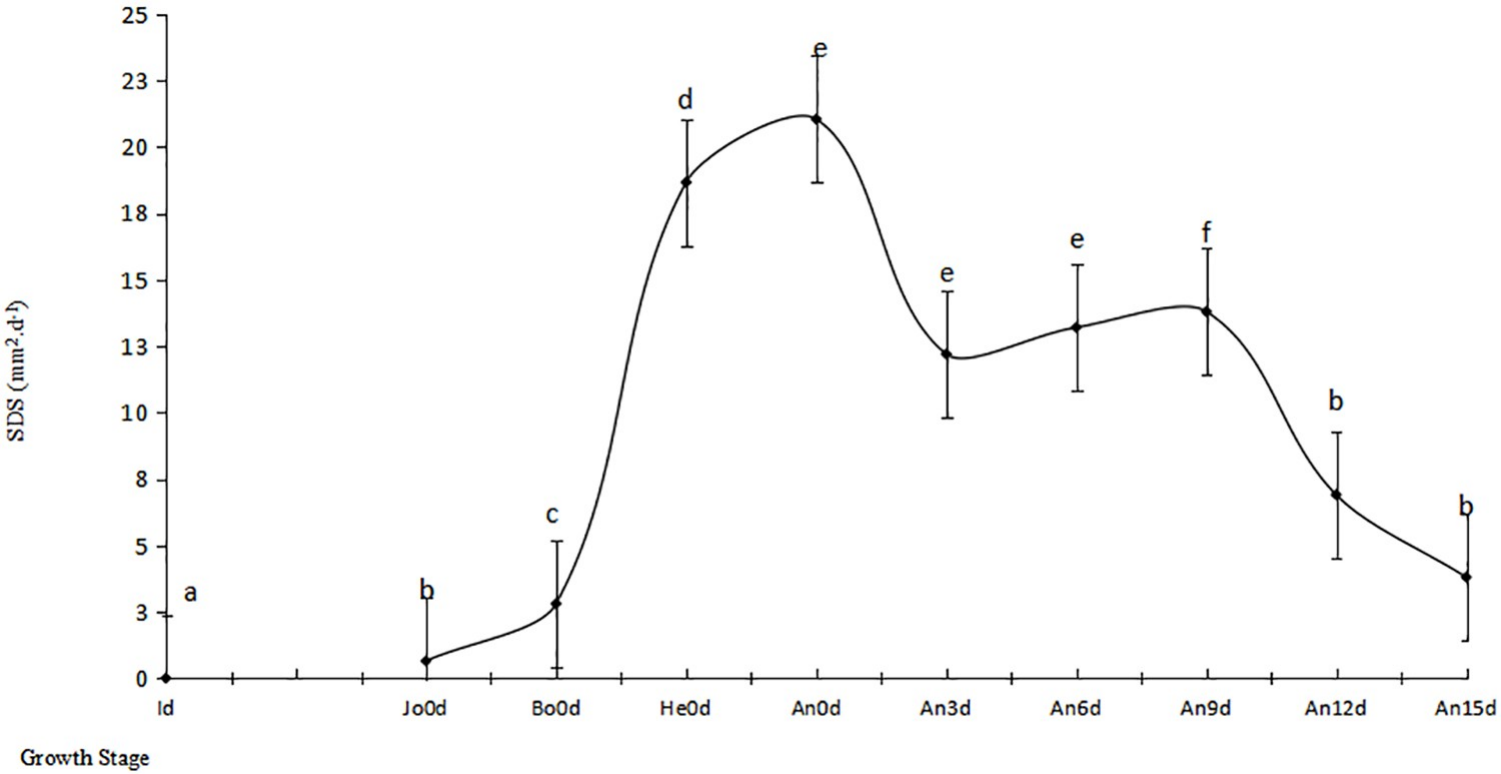
Dynamic of disease speckles speed. Note: Id was the first time of inoculation.

In order to precisely calculate the lost rate of leaf photosynthesis at the specific time, we conducted the fix leaf location experiment. The results showed IRLNC extraordinarily significantly increased from 0.65 mm^2^·d^-1^ to 2.8 mm^2^·d^-1^ (P<0.01) by 330.77% (P<0.01) from Jo0d to Bo0d, and increased from 18.66 mm^2^·d^-1^ at heading to 21.04 mm^2^·d^-1^ at anthesis by 12.75% (P<0.05) which rate was the most quickly rate of IRLNC (Fig 3, S1 Table).

The IRLNC increased from 12.2 mm^2^·d^-1^ to 13.8 mm^2^·d^-1^ from An3d to An9d (P>0.05) and decreased significantly by 44.93% (P<0.01) after An12d. The IRLNC decreased to3.8 mm^2^·d^-1^ at An15d because of too large necrosis areas and few of fresh uredinum on leaves.

### 2 The effects on graining filling (GF) processing infected by Pst

**Fig 4:**
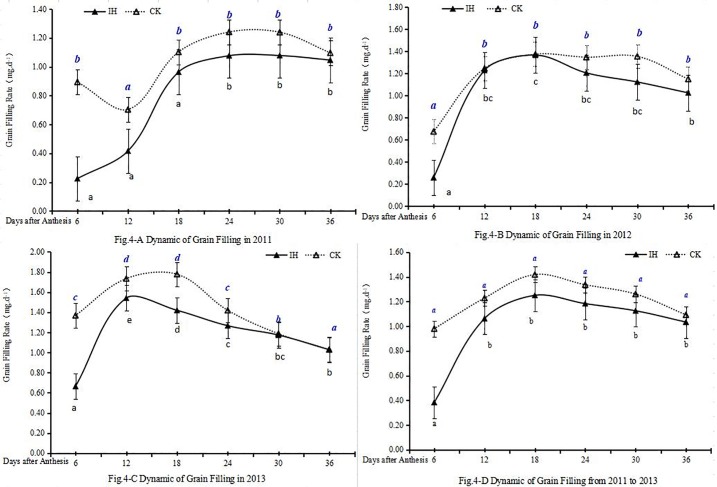
The grain filling rate dynamics of IH and CK in different season crops (4-A, 4-B, 4-C and 4-D mean in 2011, 2012, 2013 season crops and the average rate from 2011 to 2013 season crops). (Note: The letters of the dynamic line mean there were significant differences at the level of 0.05).

In 2011 year (Fig 4-A, S2 Table), the results showed grain filling rate (GFR) of IH and CK was characterized by S-type distribution. IH GFR significantly increased from 0.23 mg·d^-1^ at An6d to 0.96 mg·d^-1^ at An18d and kept 1.05 mg·d^-1^~1.08 mg·d^-1^ from An18d to An36d. CK decreased a little from 0.90 mg·d^-1^ at An6d to 0.70 mg·d^-1^ (P>0.05) at An12d, while keeping 1.10 mg·d^-1^~1.24 mg·d^-1^ from An18d to An36d. But the whole average GFR of CK was significantly higher than that of IH by 26.52% (P<0.05).

In 2012 year (Fig 4-B, S2 Table), from An6d to An 12d, GFR of IH and CK were all increased significantly from 0.26 mg·d^-1^ to 1.23 mg·d^-1^ and 0.68 mg·d^-1^ to 1.25 mg·d^-1^. IH GFR was from 1.03 mg·d^-1^ at An18d to 1.37 mg·d^-1^ at An 36d while CK was from 1.15 mg·d^-1^ to 1.25 mg·d^-1^. The average GFR of IH during whole grain filling stage was significantly slower than that of CK by 17.03% (P<0.05).

In 2013 year (Fig 4-C, S2 Table), from An6d to An12d, GFR of IH and CK were all increased significantly from 0.66 mg·d^-1^ to 1.54 mg·d^-1^ and 1.37 mg·d^-1^ to 1.73 mg·d^-1^, respectively. While after An12d, both GFR of IH and CK decreased to 1.03mg·d^-1^ at An 36d. The average GFR of CK during whole grain filling stage was significantly higher than that of IH by 15.79% (P<0.05).

In 2012 and 2013 years (Fig 4-B and 4-C, S2 Table), GFR of IH and CK dynamic was characterized by near normal distribution. The maximum of IH and CK in 2012 year were at An18d while in 2013 year IH GFR was lower but significantly 6 days earlier than that of CK (P<0.05).

The results of average GFR from 2011 to 2013 year (Fig 4, S2 Table) showed that the GFR of IH and CK characterized by the near normal distribution and their maximum were all at An18d (Fig 4-D, S2 Table). The GFR of IH extraordinarily significantly increased from 0.38 mg·d^-1^ of An6d to 1.25 mg·d^-1^ of An18 while CK from 0.98 mg·d^-1^ to1.42 mg·d^-1^. After An18d, GFR of IH and CK all kept almost constant speed from 1.03 mg·d^-1^ to 1.25 mg·d^-1^ and from 1.09 mg·d^-1^ to 1.42 mg·d^-1^, respectively. The average GFR of CK during whole grain filling stage was significantly higher than that of IH by 19.78% (P<0.05), which means stripe rust could decrease GFR remarkably.

### 3 The characteristics of thousand-kernel weight (TKW) under Pst infection

#### 3.1 The dynamic changes of TKW difference between IH and CK

Our results showed (Table 2, S2 Table) that the difference between IH and CK became smaller with the development of growth and grain filling. The three-year TKW of CK was more than that of IH by 4.56% (P>0.05), 10.87% (P>0.05) and 3.13% (P>0.05) from 2011 year to 2013 year.

**Table 2:** The TKW dynamic difference of IH and CK among different crop seasons unit: %.

Days after anthesis (d)	2011 Crop season	2012 Crop season	2013 Crop season	Average
6	-74.86a	-61.73a	-51.42a	-62.67a
12	-40.83a	-1.34b	-11.16bc	-17.78b
18	-12.59b	-0.61b	-20.14b	-11.11c
24	-13.26b	-10.54b	-10.76bc	-11.52c
30	-13.04b	-17.10b	-1.12c	-10.42c
36	-4.56b	-10.87b	-3.13c	-6.18d
Average	-26.52A	-17.03B	-16.29B	-18.98B

Note: Average line represents the annual average speed difference between grouting stage, and the rest of the column data on behalf of the anthesis within the year after different grain filing stage difference. The different letter means the differences at a = 0.05 level.

#### 3.2 The thousand-kernel weight and loss rate

The analysis of variance showed that the independent effect of Pst and the interaction with different crop seasons were not significant on TKW (Table 3, S2 Table), which proofed the leaf function lost in different seasons hardly could affect grain filling efficiency markedly. The independent effect of crop seasons appeared markedly on TKW because the drought and temperature were the most important factors during growth.

**Table 3:** The tests of between-subjects effects among different treatments.

Source	df	Mean square	Standard Erro	F Valuble	P Valuble
		**TKW**			
Tr.	3	4.771	0.571	1.218	0.335
Cr.S	1	26.861	0.808	6.859	0.019
Tr.* Cr.S	3	5.035	1.143	1.286	0.313
		**TKWL %**			
Cr.S	3	51.425	1.120	0.954	0.460

Note: R^2^ = 0.473, Adjust R^2^ = 0.243; Tr. means Treatments; Cr.S means Crop season; Tr.

* Cr.S means the interaction of treatments and crop seasons.

The thousand-kernel weight lost (TKWL) is a very important factor to evaluate the effect without leaf function during the dough stage. The TKWL (Table 4, S2 Table) of IH was less than that of CK by 4.56% (P>0.05), 10.87% (P>0.05) and 3.13% (P>0.05) in 2011, 2012 and 2013 year, respectively. And the three-year average TKWL (6.33%, P>0.05) was not significant. The results showed that Huixianhong has a special way to keep normal grain filling and almost normal TKW although leaf photosynthesis was lost at the earlier grain filling stage.

**Table 4:** The TKWL LSD difference results of IH and CK.

Crop seasons I	TWL (%)	Crop seasons II	Mean Difference	Std. Erro	P	95% Confidence Space	
						Low Bound	Up Bound
2011	4.56	2012	5.94	5.996	0.351	-7.887	19.767
		2013	-4.14	5.996	0.509	-17.971	-7.887
		2011–2013	0.86	5.996	0.890	-12.971	14.684
2012	10.87	2011	-5.94	5.996	0.351	-19.767	7.887
		2013	-10.08	5.996	0.131	-23.911	3.744
		2011–2013	-5.08	5.996	0.421	-18.911	8.744
2013	3.13	2011	4.143	5.996	0.509	-9.684	17.971
		2012	10.08	5.996	0.131	-3.744	23.911
		2011–2013	5.00	5.996	0.429	-8.827	18.827
2011–2013 Average	6.19	2011	-0.857	5.996	0.890	-14.684	12.971
		2012	5.08	5.996	0.421	-8.744	18.911
		2013	-5.00	5.996	0.429	-18.827	8.827

Note: The table showed the Least Significant Difference results of IH and CK about the TKWL.

The stripe rust could make the IH maximum speed up the rate of grain filling in advance and earlier than that of CK by 5 days, 3 days and 2 days in 2011, 2012 and 2013 year, respectively (Table 5, S3 Table). In order to assure grain filling normal and keep maximum TKW, Huixianhong produced special physiology mechanism of higher and earlier grain filling rate which could increase TKW and grain filling efficiency. At the maximum rate (Table 5, S3 Table), the IH TKW was more than that of CK by 3.38% (P>0.05) and 3.09% (P>0.05) in 2011 and 2012 year while less than by 7.84% (P>0.05) in 2013 year. And the three-year average was less than CK by 1.48% (P>0.05). The results could explain why the stage of heading and mature of IH were prior to those of CK by 2 to 4 days and 3 to 6 days, respectively.

**Table 5:** The TKW equation and max values.

Crop seasons	TrS	Equations	V_max_ (mg·TKW^-1^·d^-1^)	T_max_ (d)	TWKm (g)	TWKe (g)	TKWs (g)
2011	IH	W_11IH_ = e^4.188–24.507/T^	1.23	19	18.14	65.89	37.70
	CK	W_11CK_ = e^3.924–14.827/T^	1.33	14	17.55	50.60	39.50
2012	IH	W_12IH_ = e^4.361–22.875/T^	1.55	18	21.98	78.34	36.90
	CK	W_12CK_ = e^4.190–16.954/T^	1.61	15	21.32	66.02	41.40
2013	IH	W_13IH_ = e^4.113–16.035/T^	1.59	14	19.45	61.13	37.02
	CK	W_13CK_ = e^3.978–11.144/T^	1.63	12	21.10	53.41	37.07
2011–2013	IH	W_11-13IH_ = e^4.196–20.094/T^	1.48	16	18.92	66.42	37.21
	CK	W_11-13CK_ = e^4.028–13.949/T^	1.58	13	19.20	56.15	39.32

Note: TrS = Treatments. Vmaxe = the expected graining filling rate, W = TKW, T = days after Anthesis, T_max_ = time at the maximum grain filling rate, TKWm = TKW at the maximum grain filling rate, TKWe = the expected maximum TKW, TKWs = the sample TKW.

The growth stage showed the grain filling under the condition infected by Pst finished earlier than that under the condition without infection, which special physiological mechanism could assure the maximum plumpness of seeds and the minimum TKWL. Based on the experiment data, we built the model and equation about the grain filling rate and increased TKW after anthesis. This is the first model to simulate the process of grain filling and solve the eigenvalues under the condition infected by Pst.

In order to analyze the mechanism of grain filling, we built TKW equation based on the three-year data. The equation showed the expected TKW(TKWe) and time at the maximum rate (T_max_) of IH should be higher than those of CK (Table 5, S3 Table). It indicated that stripe rust could affect grain filling through photosynthetic function and matter transition. At the beginning of infection, in order to assure the normal grain filling, IH could produce and accumulate biomass which be transited in the other organs. It would be beneficial to TWK while leaf lost photosynthetic function. Although the IH TKWe was higher than CK, in practice, for many factors, the TKWs of IH was less than that of CK.

## Discussion

Knowledge of crop physiology in terms of grain yield determination enhances the efficiency of crop management programs. Decisions about fungicide applications during grain filling would also be based on knowledge of crop physiology. If source limitation takes place during the active grain-growth phase, grain weight could be affected by source limitation to growing grains[12]. Conversely, if the diseases affect the assimilate supply during the lag phase, grain weight potential could be affected without effects on assimilation supply [12].

The leaf function of Huixianhong was completely lost in the middle and earlier stage of grain filling, and showed similar response characteristics to stripe rust, i.e., susceptibility, high sensitivity, rapid onset, reoccurrence, rapid prevalence, rapid green loss of leaves and serious damage to leaf photosynthesis in the early stage of grain filling. This is consistent with many studies[2,4,13].

Many researchers found the incubation period of slowing rust varieties was 10.0 d-20.0 d longer than that of the corresponding control, while the latent breeding period of long-lasting stripe rust resistant varieties could reach 14.85 days.

Huixianhong's characteristics are highly susceptible, recurrent and rapid epidemic. From the first inoculation stage to the symptomatic stage, the average latent period was 13.5d in three years, which was different from the characteristics of high-susceptible, fast-developing and relapsing varieties. A longer latent period was conducive to the synthesis and accumulation of dry matter in the early stage, and it provided sufficient conditions for the transfer of dry matter to other vegetative organs. Many researchers have found that the long incubation period and the infection of stripe rust have the effect of promoting the rate of dry matter synthesis [2,14–18]. A similar conclusion was obtained in this study: the infection of stripe rust could significantly promote the synthesis and transfer rate of dry matter at the early stage.

Immune and high resistant varieties could prolong the green state of leaves to the maximum extent. It was beneficial to the early accumulation of photosynthesis and photosynthetic substances, and the plumpness and grain weight of grain were generally affected when the leaf photosynthetic ability was damaged by Pst with grain dried, thousand-kernel weight significantly reduced, yield seriously reduced, population yellow abnormal, plant, stem, leaf sheath or panicle abnormal green dry or dead[5,19–23].

However, Huixianhong showed a completely different result. After the lost of leaf source, the grain filling was still normal, the grain plumpness and the loss rate of thousand-kernel weight were lower, which was different from all the varieties previously reported. Distribution of photosynthetic substances in leaves before and after anthesis was studied. As a result, the assimilates were used in organ construction and about 3.0%-30% of which were transported to grain before anthesis. After anthesis, the assimilates contributed to grain yield significantly. Seed growth will be more dependent on pre-anthesis storage. Canopy leaf (flag leaf and upper second leaf) is the main source of dry matter, grain weight per ear is mainly determined by photosynthetic product of canopy leaf at late stage, and grain development mainly depends on photosynthesis of green organ after anthesis, especially the accumulation of photosynthesis in the upper trefoil, panicle and subear internodes[2,10,21,24–26].

Previous studies on disease index and TKWLR showed the DI of slow rust varieties was between 0.0067–0.5900 and the TKWLR was 0.08–22.350 and highly slow rust varieties with disease index 0.0152–0.0433 was still 1.03% 4.35%[17]. Some researchers also found that the different cultivars under different inffection condition have different TKWLR [7,14,15,18].

The early ending of grain filling caused by infection of stripe rust was a physiological mechanism to ensure the lowest loss rate of grain filling and TKW, which was consistent with the conclusion of many reports that other diseases lead to early maturation of crops. Huixianghong had ability to significantly reduce the loss rate of thousand-kernel weight and maintain the normal grain filling. The maximum grain filling rate was 4.75% lower than that of CK (P < 0.05), which was related to the decrease of dry matter provided for grain formation after the loss of leaf source and the main energy source for grain morphogenesis[22,24,25].

## Conclusions

Huixianhong characteristics are susceptible, highly susceptible, recurrent and rapid epidemic types of stripe rust. The leaf source of Huixianhong has lost at the early stage of grain filling due to the infection of stripe rust. Because of its own special grain filling mechanism, this variety could guarantee grain filling normally and maintain a low loss rate of thousand-kernel weight, and it is helpful for screening in stripe rust resistant breeding materials.

## Supporting information

S1 Table Original data of SE,IR,LWAR,DI and SDS (Fig 1,2 and 3). (XLSX)

S2 Table Original data of GF,TKW,TKWL,TKWLR (Fig. 4,5 Table 1-4). (XLSX)

S3 Table Original data of TKW equation (Table 5). (XLSX)
